# Maternal Carriage in Late-Onset Group B *Streptococcus* Disease, Italy

**DOI:** 10.3201/eid2709.210049

**Published:** 2021-09

**Authors:** Alberto Berardi, Caterina Spada, Roberta Creti, Cinzia Auriti, Lucia Gambini, Vittoria Rizzo, Mariagrazia Capretti, Nicola Laforgia, Irene Papa, Anna Tarocco, Angela Lanzoni, Giacomo Biasucci, Giancarlo Piccinini, Giovanna Nardella, Giuseppe Latorre, Daniele Merazzi, Laura Travan, Maria Letizia Bacchi Reggiani, Lorenza Baroni, Matilde Ciccia, Laura Lucaccioni, Lorenzo Iughetti, Licia Lugli

**Affiliations:** Azienda Ospedaliero–Universitaria Policlinico, Modena, Italy (A. Berardi, C. Spada, L. Lucaccioni, L. Iughetti, L. Lugli);; Istituto Superiore di Sanità, Rome, Italy (R. Creti); Ospedale Pediatrico Bambino Gesù, Rome (C. Auriti);; Azienda Ospedaliero-Universitaria Policlinico, Parma, Italy (L. Gambini); Ospedale Civile M. Bufalini, Cesena, Italy (V. Rizzo);; Azienda Ospedaliero–Universitaria S. Orsola–Malpighi, Bologna, Italy (M. Capretti, M.L. Bacchi Reggiani);; Ospedale Policlinico, Bari, Italy (N. Laforgia);; Ospedale Infermi, Rimini, Italy (I. Papa); Azienda Ospedaliero–Universitaria S. Anna, Ferrara, Italy (A. Tarocco);; Ospedale Santa Maria della Scaletta, Imola, Italy (A. Lanzoni);; Ospedale G. da Saliceto, Piacenza, Italy (G. Biasucci);; Ospedale Santa Maria Delle Croci, Ravenna, Italy (G. Piccinini);; Azienda Ospedaliero–Universitaria Ospedali Riuniti, Foggia, Italy (G. Nardella);; Ospedale F. Miulli, Acquaviva delle Fonti, Italy (G. Latorre);; Ospedale Valduce, Como, Italy (D. Merazzi); I; RCCS Materno Infantile Burlo Garofolo, Trieste, Italy (L. Travan);; Arcispedale Santa Maria Nuova, Reggio Emilia, Italy (L. Baroni);; Ospedale Maggiore, Bologna (M. Ciccia)

**Keywords:** Group B *Streptococcus*, sepsis, newborn, late-onset disease, bacteria

## Abstract

We retrospectively investigated mother-to-infant transmission of group B *Streptococcus* (GBS) in 98 cases of late-onset disease reported during 2007–2018 by a network in Italy. Mothers with full assessment of vaginal/rectal carriage tested at prenatal screening and at time of late onset (ATLO) were included. Thirty-three mothers (33.7%) were never GBS colonized; 65 (66.3%) were vaginal/rectal colonized, of which 36 (36.7%) were persistently colonized. Mothers with vaginal/rectal colonization ATLO had high rates of GBS bacteriuria (33.9%) and positive breast milk culture (27.5%). GBS strains from mother–infant pairs were serotype III and possessed the surface protein antigen Rib. All but 1 strain belonged to clonal complex 17. GBS strains from 4 mother–infant pairs were indistinguishable through pulsed-field gel electrophoresis. At least two thirds of late-onset cases are transmitted from mothers, who often have vaginal/rectal carriage, positive breast milk culture, or GBS bacteriuria, which suggests heavy maternal colonization.

## Abstract 2

At the time of late-onset disease, mothers often have positive breast milk culture or group B S*treptococcus* bacteriuria, suggesting heavy maternal colonization.

Group B *Streptococcus* (GBS; *Streptococcus agalactiae*) is a notable cause of sepsis and meningitis in infancy (*1*). Intrapartum antimicrobial prophylaxis (IAP) has substantially reduced the rates of early-onset disease (EOD; onset on day 0–6 postpartum) (*2*,*3*) but does not prevent late-onset disease (LOD; onset on day 7–89 postpartum) (*4*). Thus, in some settings, LOD has become the most common manifestation of neonatal GBS disease (*2*,*3*).

Prevention efforts are hampered by poor knowledge of both the pathogenesis of LOD and the relevance of any mode of GBS transmission. GBS can be transmitted from a mother to the neonate during passage through the birth canal or from sources other than delivery (*2*). A controversial issue concerns the transmission of GBS from a mother to the neonate in the postpartum period (*5*,*6*); because IAP does not eradicate maternal colonization (*5*,*7*), the mother remains a possible source of GBS transmission to the infant. The transmission of LOD GBS has been poorly investigated. Mothers of neonates with LOD show prenatal vaginal/rectal (VR) colonization ranging from 30% to 38% (*8*,*9*). However, also knowing the maternal VR status at the time of disease onset can help define the maternal carriage more precisely (*10*); this status may vary over time or, in some cases, be falsely negative at the time of screening (*11*). Breast milk has been suggested as a possible source of LOD, but its role remains controversial (*10*,*12*–*14*). It is not yet clear whether breast milk leads to LOD through repeated GBS transmission and persistent intestinal colonization (*13*) or is a marker for high levels of neonatal nasopharyngeal GBS colonization (*5*). Establishing the role of breast milk is necessary because ending breast-feeding can have long-term consequences. The literature concerning breast milk–associated cases of LOD is based almost exclusively on case reports, and we found no studies in large populations that provide stronger evidence. Finally, quantifying the burden of LOD transmitted from mothers can help in predicting the effects of future strategies, because a GBS vaccine might reduce maternal carriage (*15*).

To clarify the dynamics of GBS mother-to-infant transmission, we defined maternal carriage on the basis of VR status assessed both at the prenatal screening and at the time of disease onset with full assessment of maternal carriage. We used additional maternal cultures collected from urine and breast milk at disease onset to investigate further possible associations with neonatal LOD.

## Methods

We retrospectively analyzed data from a network of hospitals in Italy. Episodes of LOD GBS are anonymously reported on a monthly basis to the coordinating center, Azienda Ospedaliero—Universitaria of Modena (Modena, Italy) (*10*). Hospitals participating in the network follow US Centers for Disease Control and Prevention (CDC) guidelines regarding antenatal GBS screening and IAP administration to women who are GBS-colonized (*11*). During January 1, 2007–December 31, 2018, we received notification of 175 cases of LOD, of which 98 had a full assessment of maternal carriage (see definitions in Appendix). We used a special form for surveillance, designed for both EOD and LOD reporting, that included patient demographics, mode of delivery, risk factors for EOD, and IAP administration. Surveillance officers extracted all clinical information from the labor and delivery records using this standardized form; they obtained any missing data by telephone from the coordinating center. To maintain patient confidentiality, spreadsheets submitted to the principal investigator were anonymous and did not include any identifiable data of patients or caregivers. The case reporting and isolate collection were determined to be non-research public health surveillance. The local ethical committee of Azienda Ospedaliero–Universitaria of Modena approved the study (no. 423/2019). We obtained a waiver of informed consent for each of the patients included.

### Microbiological Methods

We processed vaginal and rectal samples according to CDC recommendations: growth in preenrichment broth and isolation in selected media. We collected and cultured breast milk samples as previously described (*10*). We processed blood, cerebrospinal fluid, and urine cultures with automated systems, Bactec 9240 (Becton Dickinson, https://www.bd.com) and Bactalert (bioMérieux, https://www.biomerieux.com).

We sent the maternal and infant LOD GBS strains isolated at the time of onset to the National Reference Center for Streptococci at Istituto Superiore di Sanità (Rome, Italy). We performed species confirmation by determining group B Lancefield surface antigen using the Streptococcal Grouping kit (Oxoid, https://www.oxoid.com). We based serotyping on a commercial latex agglutination test, ImmuLex *Streptococcus* Group Kit (SSI Diagnostica, https://www.ssidiagnostica.com). We performed molecular typing of capsular types Ia-IX using a multiplex PCR assay (*16*); we identified surface protein antigens belonging to the α-like family by a multiplex PCR (*17*). We assessed bacterial genetic population structure by multilocus sequence typing (MLST) and, for selected strains, by pulsed-field gel electrophoresis (PFGE). We assessed antimicrobial susceptibility profile to erythromycin, clindamycin, and tetracycline as previously described (*18*,*19*). We identified pilus island gene content using PCR (*20*).

### Maternal Cultures

We tested GBS carriage at the vaginal and rectal sites both at the prenatal screening and at the time of LOD onset in a full assessment of maternal carriage. We collected additional breast milk and urine cultures from mothers at LOD onset. We conducted molecular analyses on the available maternal GBS strains collected at the time of LOD onset.

### Statistical Analyses

We used Stata/SE version 14.2 (StataCorp, https://www.stata.com) and MedCalc version 9.3 (MedCalc Software, https://www.medcalc.org). Continuous variables are expressed as mean +SD or median and interquartile range (IQR), and categorical data are expressed as numbers (percentages). We compared categorical and continuous variables across patient groups using a χ^2^ test, Fisher exact test, Student t-test, or Mann-Whitney test, as appropriate. All p values refer to 2-tailed tests of significance; p<0.05 was considered significant.

## Results

A full assessment of maternal carriage was available in 98 cases of LOD during 2007–2018. Most cases of LOD (89/98) came from a regional area-based surveillance in which incidence of EOD is 0.18/1,000 live births (*21*) and of LOD is 0.31/1,000 live births (*10*), and the prevalence of maternal VR colonization is 21% (*22*).

Eighty (81.6%) cases occurred in full-term neonates and 18 (18.4%) in preterm neonates (of which 10 were still in hospital at the time of LOD onset). Compared with full-term neonates, preterm neonates were less likely to be delivered vaginally and more likely to undergo mechanical ventilation (Table 1). Twenty mothers (3 preterm and 17 full-term) were exposed to adequate IAP; of those, 17 (85%) carried GBS at the time of LOD diagnosis.

**Table 1:** Demographics and clinical data of neonates with late-onset group B *Streptococcus* disease, Italy*

Characteristic	LOD cases in preterm neonates, n = 18†	LOD cases in full-term neonates, n = 80	p value	Total, N = 98
Median birthweight, g (IQR)	1,285 (987–1,800)	3,185 (2,898–3,518)	NA	3,110 (2,570–3,425)
Gestational age at delivery, wks, median (IQR)	31.0 (27.0–33.0)	39.0 (38.0–40.0)	NA	39 (38–40)
Vaginal delivery	4 (22.2)	56 (70.0)	<0.01	60 (61.2)
Planned caesarean section	8 (44.4)	18 (22.5)	0.11	26 (26.5)
IAP exposure‡	9 (50.0)	29 (36.3)	0.42	38 (38.8)
Age at onset, median, d (IQR)	33 (26–45)	27 (15–43)	0.08	29 (16–43)
Mechanical ventilation	7 (38.9)	4 (5.0)	<0.01	11 (11.2)
Focal infection§	0	6 (7.5)	0.51	6 (6.1)
Meningitis with or without sepsis¶	8 (57.1)	32 (56.1)	0.82	40 (56.3·)
Brain lesions at discharge from hospital#	7 (38.9)	15 (18.8)	0.12	22 (22.4)
Death	1 (5.6)	1 (1.3)	0.81	2 (1.0)

*Values are no. (%) except as indicated. GBS, group B streptococcus; IAP, intrapartum antibiotic prophylaxis; IQR, interquartile range; LOD, late-onset disease; NA, not applicable. †14 were early to moderate and 4 were late preterm neonates. ‡IAP was adequate (ampicillin, penicillin, or cefazolin given >4 h before delivery) in 3/9 (33.3%) cases in preterm neonates and in 17/29 (58.6%) cases in full-term neonates. §GBS-positive blood culture result associated with focal signs outside the respiratory tract (cellulitis, arthritis, parotiditis, others) (*10*). ¶Percentage and significance were calculated based on findings from lumbar puncture (in preterm neonates, 14 cases; in full-term neonates, 57 cases). Meningitis was culture-proven in 36/40 cases. #Brain lesions were confirmed through ultrasound, magnetic resonance study, or both.

Thirty-three (33.7%) of 98 mothers were persistently not GBS-colonized; the other 65 (66.3%) mothers were GBS-colonized, 36 (36.7%) persistently (Table 2). Maternal VR colonization was more likely to be detected at the time of onset (58/65) than at the prenatal screening (43/65; p<0.01). At the time of LOD onset, 59.2% of mothers were colonized, 18.9% had asymptomatic GBS bacteriuria, and 20.5% had positive breast milk culture. 

**Table 2:** Maternal cultures of group B *Streptococcus*, Italy*

Culture	Preterm cases, n = 18	Missed cases	Full-term cases, n = 80	Missed cases	p value	Total cases, n = 98
Cultures before delivery						
VR tested	18 (100)	0	80 (100)	0	NA	98 (100)
Positive culture	8 (44.4)	0	35 (43.8)	0	0.83	43 (43.9)
Urine tested	18 (100)	0	74 (92.5)	6	0.51	92 (93.9)
GBS bacteriuria†	0 (0)	0	2 (2.7)	0	0.85	2 (2.2)
Cultures at onset of LOD						
VR tested	18 (100)	0	80 (100)	0	NA	98 (100)
Positive culture	7 (38.9)	0	51 (63.8)	0	0.09	58 (59.2)
Urine tested	18 (100)	0	72 (90.0)	8	0.36	90 (91.8)
GBS bacteriuria†	5 (27.8)	0	12 (16.7)	0	0.46	17 (18.9)
Breast milk tested‡	15 (83.3)	3	68 (85.0)	12	0·85	83 (84.7)
Positive culture†	2 (13.3)	0	15 (22.1)	0	0.69	17 (20.5)

*Values are no. (%) except as indicated. Samples were obtained during pregnancy (urine), at prenatal screening (VR), and at onset of LOD (urine, VR, and breast milk). GBS, group B *Streptococcus*; LOD, late-onset disease; NA, not assessable; VR, vaginal/rectal. †Percentage and significance are calculated based on the patients who were tested. ‡Among 18 preterm neonates, 7 were exclusively fed breast milk (1 positive culture), 7 were fed pump-extracted milk (1 positive culture), 2 were fed formula milk, 1 was fed mixed breastmilk (missing culture), and 1 was fed donor human milk. Among 80 full-term neonates, 58 were exclusively fed breast milk (14 positive cultures and 1 missing culture), and 10 were fed mixed breast milk (1 positive culture and 1 missing culture).

All mothers with asymptomatic GBS bacteriuria also carried GBS at the VR site. Median urinary bacterial count was 40,000 CFU/mL (interquartile range [IQR] 10,000–100,000 CFU/mL; range 1,000–1 million CFU/mL). GBS bacteriuria was significantly more likely to be detected at the time of LOD onset (17/90 tested) rather than during pregnancy (2/92 tested; p<0.01).

Among 17 women with a positive breast milk culture, 1 mother had mastitis (1 million CFUs/mL) and 16 had no mastitis (median bacterial count 300,000 CFU/mL; IQR 100,000–725,000 CFU/mL; range 9,000–6,400,000 CFU/mL). Fourteen (82.4%) of the 17 mothers were GBS colonized at the VR site at the prenatal screening or at the time of onset, or both, but the other 3 (17.6%) were persistently not colonized.

### Urine and Breast Milk Cultures According to Maternal VR Carriage

**Figure 1:**
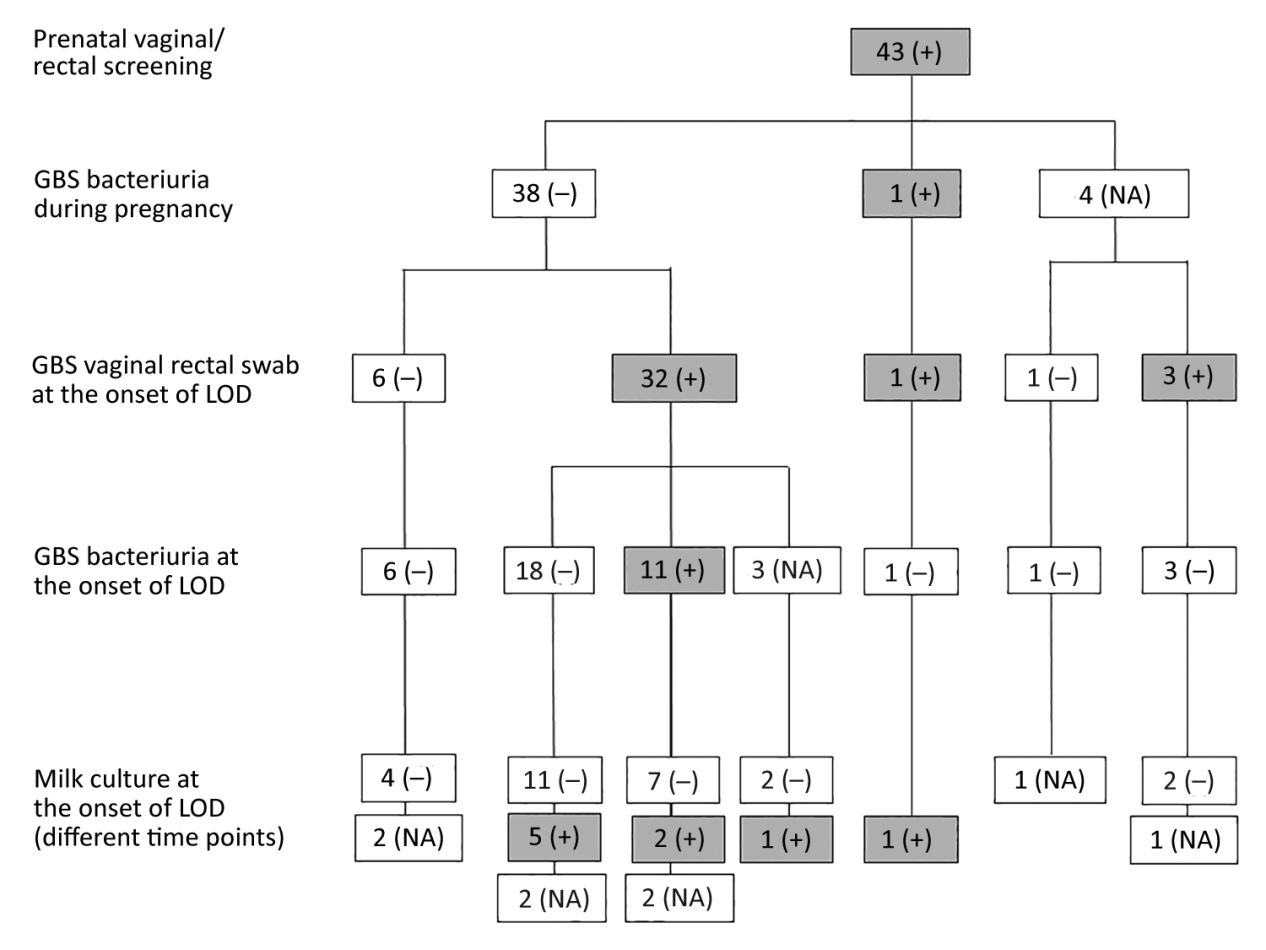
Longitudinal analysis of cultures obtained from women carrying GBS at the vaginal/rectal site at screening. Gray-shaded boxes represent GBS positivity. GBS, group B *Streptococcus*; LOD, late-onset disease; NA, not assessed; –, negative; +, positive.

Forty-three women tested GBS colonized at the VR site at the prenatal screening (Figure 1). At the time of LOD onset, most (36, 83.7%) were confirmed GBS-colonized at the VR site; of those, 11/34 tested (32.4%) also had GBS bacteriuria and 9/32 tested (28.1%) had positive breast milk culture.

**Figure 2:**
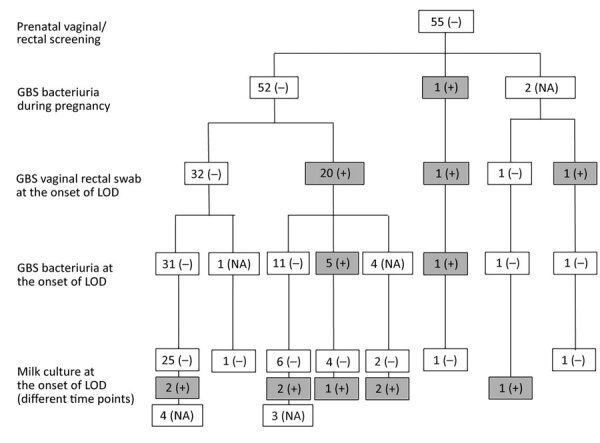
Longitudinal analysis of cultures obtained from women who did not carry GBS at the vaginal/rectal site at screening. GBS, group B *Streptococcus*; LOD, late-onset disease; NA, not assessed; –, negative; +, positive.

Fifty-five women tested GBS-noncolonized at the prenatal VR screening (Figure 2). At the time of LOD onset, 40% (22/55) carried GBS at the VR site; of those, 31.6% (6/19 tested) also had GBS bacteriuria and 5/19 tested (26.3%) tested positive in the breast milk culture. Overall, we found very high frequencies of GBS bacteriuria (33.4%) and GBS-positive breast milk (27.5%) in women with VR colonization at the time of LOD onset, independent of the VR status at prenatal screening.

### GBS Molecular Typing

Fifty-eight mothers were GBS colonized at the time of LOD onset, and the cultures obtained from 20 (34.5%) of them were available along with isolates from their infants for molecular typing. Overall, 57 bacterial isolates from different sources were available (Table 3). We collected 24 strains of neonatal isolates from blood, CSF, or both and collected maternal isolates from VR swab only (7 cases), milk only (3 cases), both VR swab and milk (7 cases), or both VR swab and urine (3 cases). All GBS strains were serotype III and possessed the surface protein *Rib* gene. At MLST analysis, all strains collected from mother–infant pairs were sequence type (ST) 17, which is part of the clonal complex (CC) 17 (*6*,*19*), except for 1 mother–infant pair whose strains were ST449. Each mother–infant pair displayed the same antimicrobial susceptibility profile; only strains from 3 pairs were resistant to both erythromycin and clindamycin, mediated by the *ermB* gene. Of note, these resistant strains were also resistant to tetracycline mediated by the *tetO* gene and, unlike all other strains that possessed the pili island (PI) 1 and 2b, they lacked PI-1 and had only PI-2b (Table 3).

**Table 3:** Mother-infant pairs and isolates of group B *Streptococcus* in study of late-onset disease, Italy*

Pair	Maternal isolates				Infant isolates		Sequence type	Pili island gene content	Erythromycin resistance genes				Tetracycline resistance genes	
	Urine	VR	Breast milk		Blood	CSF			*erm*B	*erm*A	*mef*A/E		*tet*M	*tet*O
Pair 1			X		X	X	ST449	1+2b	–	–	–		+	–
Pair 2		X			X		ST17	1+2b	–	–	–		+	–
Pair 3		X	X		X		ST17	1+2b	–	–	–		+	–
Pair 4			X		X		ST17	1+2b	–	–	–		–	–
Pair 5		X	X		X	X	ST17	1+2b	–	–	–		+	–
Pair 6	X	X			X		ST17	1+2b	–	–	–		+	–
Pair 7			X		X		ST17	1+2b	–	–	–		+	–
Pair 8		X			X		ST17	1+2b	–	–	–		+	–
Pair 9	X	X			X		ST17	1+2b	–	–	–		+	–
Pair 10		X	X		X		ST17	1+2b	–	–	–		+	–
Pair 11	X	X			X		ST17	1+2b	–	–	–		–	–
Pair 12		X	X		X		ST17	1+2b	–	–	–		–	–
Pair 13		X	X		X		ST17	1+2b	–	–	–		+	–
Pair 14		X			X	X	ST17	1+2b	–	–	–		+	–
Pair 15		X			X		ST17	1+2b	–	–	–		+	–
Pair 16		X			X		ST17	1+2b	–	–	–		+	–
Pair 17		X			X		ST17	2b	+	–	–		–	+
Pair 18		X			X		ST17	1+2b	–	–	–		+	–
Pair 19		X*	X†		X		ST17	2b	+	–	–		–	+
Pair 20		X	X†		X	X	ST17	2b	+	–	–		–	+

*CSF, cerebrospinal fluid; ST, sequence type; VR, vaginal/rectal; –, negative; +, positive. †Two samples were taken at different times.

In addition, we analyzed 12 bacterial isolates from 4 mother–infant pairs by PFGE. We assigned strains within each pair the same PFGE type if they presented an identical genomic band pattern profile (*18*).

## Discussion

It is crucial to understand the pathway of GBS transmission in LOD to determine the necessary interventions. We collected a large set of maternal cultures at the onset of LOD and focused on mothers with a full assessment of VR carriage. At the time of LOD onset, a substantial proportion of mothers were found to carry GBS at the VR site, although some of them were GBS-noncolonized at prenatal VR screening. Maternal GBS colonization is an important risk factor for GBS disease, and determining the extent and types of colonization is essential for the formulation of a vaccine against GBS (*8*,*23*,*24*).

Rates of maternal asymptomatic GBS bacteriuria were strikingly high (≈19%). GBS bacteriuria, which affects 2%–7% of pregnant women (*11*) (2.2% in a recent area-based study in Italy [*22*]), is a marker for heavy genital tract colonization. GBS bacteriuria is associated with an increased risk for EOD in neonates (*11*), but its role in LOD has not been previously investigated. GBS bacteriuria was present in approximately one third of the cases among mothers with VR colonization at the time of LOD onset. This observation suggests that mothers, especially those heavily colonized, may be a main source of GBS exposure for their infants. Indeed, molecular typing indicated that GBS isolates collected from mother-infant pairs were closely related. All maternal and infant bacterial strains were serotype III and possessed the surface antigen Rib, and all but 1 pair displayed the same MLST type. The common origin of the bacterial maternal–infant pairs was confirmed by PFGE analysis when performed and the comparable antimicrobial susceptibility profile. This finding is consistent with a previous longitudinal study in 160 mother–infant pairs, which demonstrated that GBS strains isolated from healthy neonates and their mothers until 8 weeks postpartum were indistinguishable (i.e., had identical band patterns) by PFGE analyses (*5*). Globally, serotype III strains are clinically the most important, accounting for 25% of colonizing strains and 62% of strains causing invasive disease in infants, although geographic variation exists (*1*).

In this study, strains from all but 1 case belonged to clonal complex (CC) 17, a hypervirulent clonal lineage predominantly responsible for both EOD and LOD. In animal models, GBS CC17 shows higher abilities of intestinal colonization and translocation through physiologic barriers (*25*,*26*); >80% of cases of GBS serotype III LOD worldwide are caused by the hypervirulent CC17 (*6*,*19*,*26*,*27*). The emergence of a multidrug-resistant CC17 sublineage has been increasingly reported since its identification in China, Canada, and Europe (*27*–*29*); it is identifiable by the replacement of the pilus island 1 genetic locus by mobile elements carrying both *tetO* and *ermB* genes plus aminoglycoside resistance genes. The presence of the *tetO-ermB* genes along with that of PI-2b alone can be considered a marker of the emerging multidrug-resistant hypervirulent CC17 clonal lineage (*27*–*29*). Although we did not perform a detailed genomic analysis for all GBS strains, the antimicrobial resistance we detected was probably due to this multidrug-resistant CC17 subclone whose dissemination is still limited among neonatal infections in Italy. GBS resistance to clindamycin is well documented, but is relevant to only a small population of women and not to infants.

Mother-to-infant postdelivery GBS transmission can be assumed in some cases. Indeed, many neonates were born to mothers who had been exposed to IAP (which interrupts maternal-to-fetal transmission). Because maternal VR carriage at the time of late onset was confirmed in 85% of mothers who received adequate IAP, a postdelivery transmission is likely. This finding is consistent with recent studies showing risks of neonatal postpartum colonization from a maternal source (*5*,*6*,*25*). The importance of maternal colonization is probably greater in neonates born full-term because have frequent and close contact with their mothers, whereas preterm neonates admitted to hospital have fewer chances for transmission of GBS during close contact with their mothers. Although in this study VR colonization rates at the time of LOD onset were higher in full-term mothers (64% vs. 39% in preterm mothers), the difference was not significant, perhaps because of the small sample size.

In this study, we found GBS in ≈20% of breastfeeding mothers. Mastitis in LOD was infrequent; mothers with positive breast milk culture were more often asymptomatic, although their milk bacterial counts were sometimes high. This finding suggests a silent maternal duct colonization, and it is consistent with case reports of GBS breast milk–associated LOD, in which most mothers have no sign of mastitis (*13*).

Furthermore, in our study most mothers with positive breast milk culture carried GBS at the VR site, which was often heavily colonized. Maternal VR carriage would appear to be associated with GBS transmission into breast milk, perhaps in some cases after translocation from the gastrointestinal tract through the lymphatic system to the mammary glands (*30*). In contrast, only 3 mothers who had positive breast milk culture were persistently GBS-noncolonized at the VR site. In such cases, a circular mechanism of GBS transmission to neonates could be implicated. The retrograde theory assumes that GBS, present in the infant’s throat, colonizes the mammary ducts during breast-feeding; GBS load increases in the milk, and, in turn, the infant is infected during breast-feeding. Our data do not suggest that breast milk itself is a risk factor for LOD. Breast milk is known to contain immunomodulatory and antimicrobial components (*12*) (i.e., sIgA and cytokines) that may protect from LOD, and the lack of these components seems to increase the risk of persistent neonatal colonization (*31*) and LOD (*32*).

Taken all together, these results show that mothers are largely the predominant source of GBS in cases of LOD, both during childbirth (especially if IAP is not given) and in the postpartum period. GBS-positive breast milk is one of the ways by which heavily colonized mothers expose their infants to GBS.

The first limitation of our investigation is that it was an observational study without a control group. Therefore, the relevance of a positive breast milk in LOD could not be clearly assessed, because we do not know how many breastfeeding GBS-colonized mothers with healthy neonates have GBS-positive breast milk. However, Berardi et al. (*5*) found much lower rates (≈7%) of GBS-positive breast milk in a cohort of breastfeeding mothers (GBS-colonized at the VR site) who had healthy neonates. In addition, we cannot rule out an accidental contamination of some breast milk samples during collection, although we had provided instructions for collection. Second, although we proposed doing so, we did not systematically perform full assessment of VR culture both at prenatal screening and at the time of disease onset; just over half of the mothers had the full assessment during surveillance. In fact, not all of them had prenatal screening; furthermore, the collection of cultures at the time of LOD diagnosis and then shipping the maternal strains were challenging to organize. Third, the PFGE analysis was available only in a few mother-infant pairs. However, previous studies demonstrate that the concordance of GBS strains collected from mothers and their own neonates at delivery or in the following weeks is very high, reaching ≈100% of cases (*5*,*6*,*18*). Finally, maternal colonization rates could be higher than we found as a result of inherent insufficient sensitivity of maternal VR cultures (*11*), which may lead to false-negative culture results. We did not investigate the possible role of the father in the transmission of GBS.

In conclusion, this study suggests that most cases of LOD are strictly associated with maternal VR colonization and that CC17 is the predominant clonal lineage. Rates of asymptomatic GBS bacteriuria at the time of LOD onset are strikingly high, and this finding suggests heavy maternal colonization. Positive breast milk culture is relatively common among asymptomatic breastfeeding mothers of neonates with LOD, especially if they carry GBS at the VR site. However, the causal role of breastfeeding remains uncertain, and our data do not lead to definitive conclusions. Mother-to-infant transmission may occur after delivery. Our findings call attention to maternal transmission after delivery as an underestimated source of neonatal LOD and may assist in predicting the impact of maternal GBS vaccination.

Appendix Additional information about maternal carriage of group B *Streptococcus* in late-onset disease, Italy.
