# Supplementary material for: Maternal Carriage in Late-Onset Group B Streptococcus Disease, Italy
Source: Emerg Infect Dis. 2021 Sep;27(9):2279–87. doi: 10.3201/eid2709.210049 (PMC8386770; doi:10.3201/eid2709.210049)
Supplement: Appendix — Additional information about maternal carriage of group B Streptococcus in late-onset disease, Italy. [file 21-0049-Techapp-s1.pdf]

Article DOI: https://doi.org/10.3201/eid2709.210049

Maternal Carriage in Late-Onset Group B *Streptococcus* Disease, Italy

Appendix

Glossary

GBS: group B *Streptococcus*.

GBS case: isolation of GBS from a normally sterile body site (e.g., blood or cerebrospinal fluid) in infants 7–89 days of age (*1*).

Maternal GBS bacteriuria: GBS found at any bacterial concentration in the urine.

GBS mastitis: localized, painful inflammation of the breast occurring in conjunction with maternal flu-like symptoms (e.g., fever) and yield of GBS in the milk culture (*2*).

Full assessment of maternal status: GBS carriage assessed at the VR site both at prenatal screening and at onset of LOD in the infant.

GBS-colonized: mothers who tested positive at the vaginal-rectal site at prenatal screening or at onset of LOD in the infant.

Persistently GBS-colonized: mothers who tested positive at the vaginal-rectal site both at prenatal screening and at onset of LOD in the infant.

Persistently GBS-noncolonized: mothers who tested negative at the vaginal-rectal site both at prenatal screening and at onset of LOD in the infant.

Sepsis: growth of GBS from blood culture associated with symptoms consistent with sepsis (*3*).

References

1. Nanduri SA, Petit S, Smelser C, Apostol M, Alden NB, Harrison LH, et al. Epidemiology of invasive early-onset and late-onset group B streptococcal disease in the United States, 2006 to 2015: multistate laboratory and population-based surveillance. JAMA Pediatr. 2019;173:224–33. PubMed https://doi.org/10.1001/jamapediatrics.2018.4826

2. Berardi A, Rossi C, Lugli L, Creti R, Bacchi Reggiani ML, Lanari M, et al.; GBS Prevention Working Group, Emilia-Romagna. Group B *Streptococcus* late-onset disease: 2003–2010. Pediatrics. 2013;131:e361–8. PubMed https://doi.org/10.1542/peds.2012-1231

3. Madrid L, Seale AC, Kohli-Lynch M, Edmond KM, Lawn JE, Heath PT, et al.; Infant GBS Disease Investigator Group. Infant group B streptococcal disease incidence and serotypes worldwide: systematic review and meta-analyses. Clin Infect Dis. 2017;65(suppl_2):S160–72. PubMed https://doi.org/10.1093/cid/cix656
